# McIndoe Vaginoplasty in MRKHS: Case Report and Literature Review

**DOI:** 10.1002/ccr3.70009

**Published:** 2024-12-15

**Authors:** Newton Ashish Shah, Manish Yadav, Suman Verma, Digraj Yadav, Aalok Kumar Yadav, Asmita Ghimire, Hima Rijal, Poonam Koirala

**Affiliations:** ^1^ Tribhuvan University Institute of Medicine Kathmandu Nepal; ^2^ Department of Obstetrics and Gynecology Tribhuvan University, Institute of Medicine Kathmandu Nepal; ^3^ Department of Radiology Tribhuvan University, Institute of Medicine Kathmandu Nepal

**Keywords:** Mayer–Rokitansky–Kuster–Hauser syndrome, MRKHS, Müllerian aplasia, Vaginoplasty

## Abstract

Mayer–Rokitansky–Küster–Hauser syndrome (MRKHS) is a rare congenital disorder characterized by agenesis of the uterus and upper two‐thirds of the vagina. It affects around 1 in 4000–5000 females and is of two types: type 1, also known as isolated Müllerian aplasia or Rokitansky, which involves only uterovaginal agenesis, and type 2, presents as uterovaginal agenesis along with renal, cardiac, and other organ anomalies. Despite the absence of vaginal and uterine structures, individuals with MRKHS typically present with normal secondary sexual characteristics and ovarian functions. A 26‐year‐old female presented with primary amenorrhea. Despite this, she had typical secondary sexual characteristics. Physical examination revealed normal pubic hair distribution and external genitalia, consistent with Tanner's stage 5 development. Further evaluation via magnetic resonance imaging (MRI) confirmed diagnosis of MRKHS type II, revealing the absence of the uterus and upper two‐thirds of the vagina, with normal ovaries and absent left kidney. A multidisciplinary team performed McIndoe vaginoplasty creating a successful neovagina, measuring 7 cm in length, using split‐thickness skin graft. MRKHS, a rare congenital anomaly involving Müllerian duct abnormalities, poses diagnostic and therapeutic challenges. The etiology remains unknown, but it likely stems from defective Müllerian fusion during embryologic development. Differential diagnosis from conditions like androgen insensitivity syndrome (AIS) is crucial. MRI is important in diagnosis, revealing associated anomalies. Treatment aims to restore sexual function. This case presents that MRKHS can occur with normal hormonal function and typical secondary sexual characteristics. Neovagina creation is an effective surgical intervention for young females seeking to restore sexual function.

**Summary**

- Mayer–Rokitansky–Küster–Hauser syndrome (MRKHS) is a rare congenital disorder characterized by the absence of the uterus and upper two‐thirds of the vagina, affecting around 1 in 4000–5000 females.
- A 26‐year‐old female presented with primary amenorrhea, absent uterus, part of the vagina, and a missing left kidney, yet displayed typical secondary sexual characteristics and normal ovarian function.
- Magnetic resonance imaging (MRI) confirmed MRKHS type II. McIndoe vaginoplasty was performed to create a neovagina.
- MRKHS presents diagnostic challenges, often diagnosed via MRI. Differential diagnosis includes androgen insensitivity syndrome. Psychological counseling is crucial because of its impact on emotional well‐being.
- Neovagina creation offers significant benefits for individuals with MRKHS, helping restore sexual function and addressing psychological concerns associated with the condition.

## 1 Introduction

Mayer–Rokitansky–Küster–Hauser syndrome (MRKHS) is a rare congenital disorder characterized by aplasia of the uterus and upper two‐thirds of the vagina. The incidence of MRKHS is approximately one in 4000–5000 females. It is classified into two types: type 1, also known as isolated Müllerian aplasia or Rokitansky, which involves only uterovaginal agenesis, and type 2, which encompasses uterovaginal agenesis along with renal, cardiac, and other organ anomalies, as well as amenorrhea and dyspareunia [1]. Individuals with MRKHS typically present with primary amenorrhea, normal secondary sexual characteristics, and a normal 46XX karyotype [2]. Despite the absence of vaginal and uterine structures, external genitalia are typically unaffected, and ovarian function remains intact [2]. The exact etiology of MRKHS is unknown. Treatment of vaginal agenesis typically involves the creation of a neovagina to facilitate normal sexual intercourse. This can be achieved through various surgical and nonsurgical techniques. Given that MRKHS is often diagnosed during adolescence, a critical period for emotional development, psychological counseling is an integral component of patient care to address concerns regarding infertility [3]. Here, we present a rare case of a 26‐year‐old female diagnosed with MRKHS type II who underwent successful vaginoplasty. We report the case adhering to SCARE guidelines 2023 [4].

## 2 Case History/Examination

A 26‐year‐old female presented with primary amenorrhea, which began 10 years ago. At the age of 15, she initially visited our outpatient department (OPD) and underwent ultrasonography (USG) which showed the absence of a uterus and a part of the vagina. Along with absence of left kidney. However, she had typical secondary sexual characteristics, including normal breast development and pubic hair distribution. She denied any cyclic abdominal pain, vaginal bleeding, headaches, visual impairments, galactorrhea, or notable weight fluctuations. There was no significant past, medical history. Her familial history revealed normal menstrual patterns in both her mother and sister, and her mother confirmed the absence of any substance use, infections, or exposure to radiation during her gestational period.

Upon examination, the patient was well‐oriented with a height of 155 cm and weight of 56 kg, appropriate for her age. Vital signs were normal, and her secondary sexual characteristics were with Tanner's stage 5 consistent for her age. Abdominal examination revealed no abnormalities, whereas genital examination showed normal pubic hair distribution, labia majora and minora, and external urethral meatus. The peripheral blood karyotype was 46XX. Laboratory parameters, including hormone levels (LH, FSH, prolactin, progesterone, and testosterone), were within normal limits. Perrectal examination did not reveal any palpable masses and the uterus was absent. Based on this history and examination, a provisional diagnosis of Müllerian agenesis was made.

## 3 Methods

**FIGURE 1:**
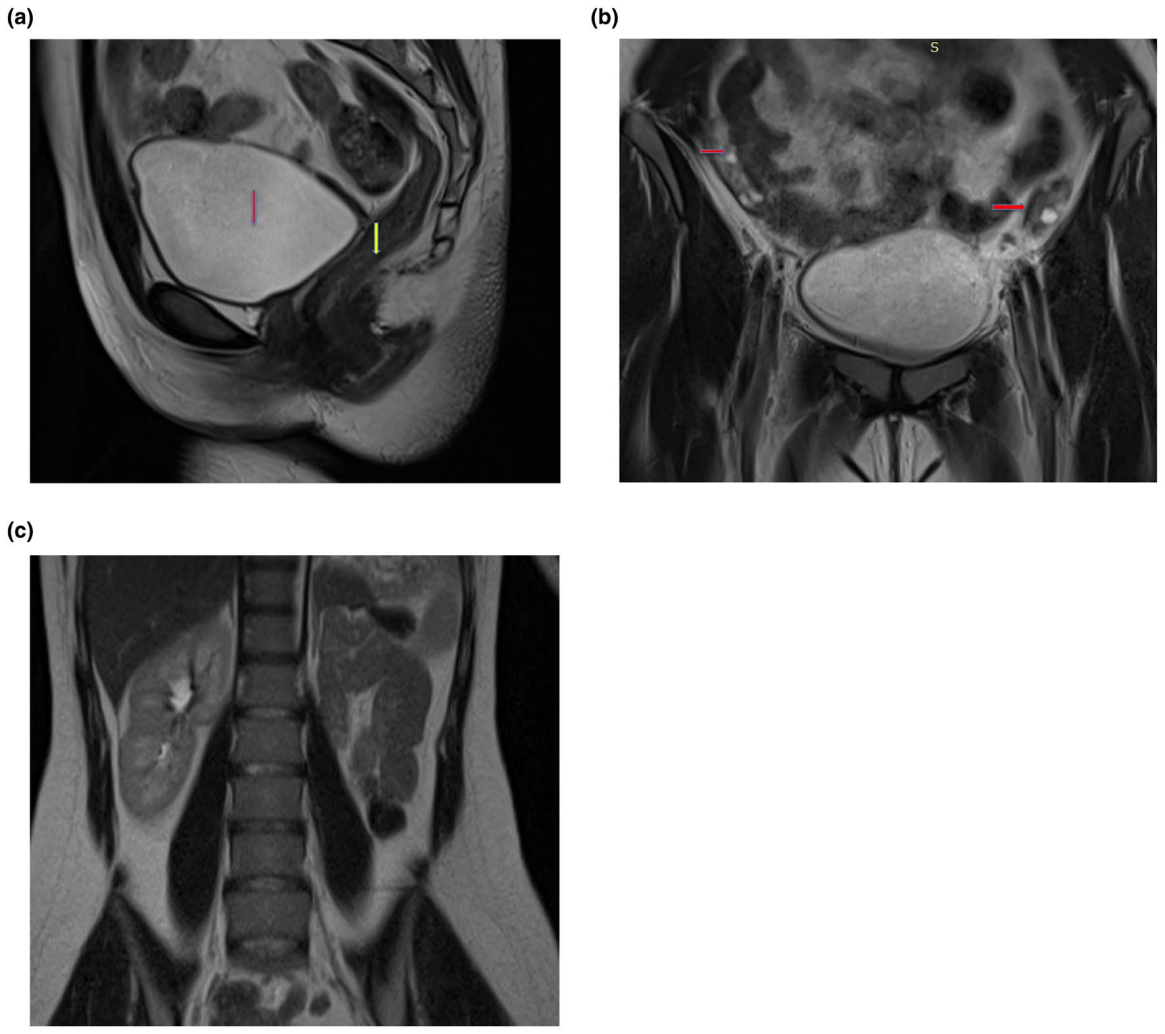
(a) T2 sagittal image of the pelvis showing urinary bladder anteriorly (red arrow) and rectum posteriorly (yellow arrow) with the absence of uterus and upper 2/3rd of the vagina. (b) T2 coronal image of the pelvis showing bilateral normal ovaries (marked by red arrows) high up in the pelvis anterior to the psoas muscle. (c) T2 Haste coronal image of the abdomen showing normal right kidney and absence of left kidney.

On Further evaluation, magnetic resonance imaging (MRI) was done which revealed the absence of the uterus and upper two‐thirds of the vagina, with a blind‐ending distal one‐third of the vagina (Figure 1). Both ovaries were located higher and more peripherally, anterior to the iliopsoas muscle, with normal size and morphology. (Figure 1b) Additionally, the left kidney was not visualized on MRI (Figure 1c). Rest all other findings were normal. Echocardiography (ECHO) screening showed normal findings.

**FIGURE 2:**
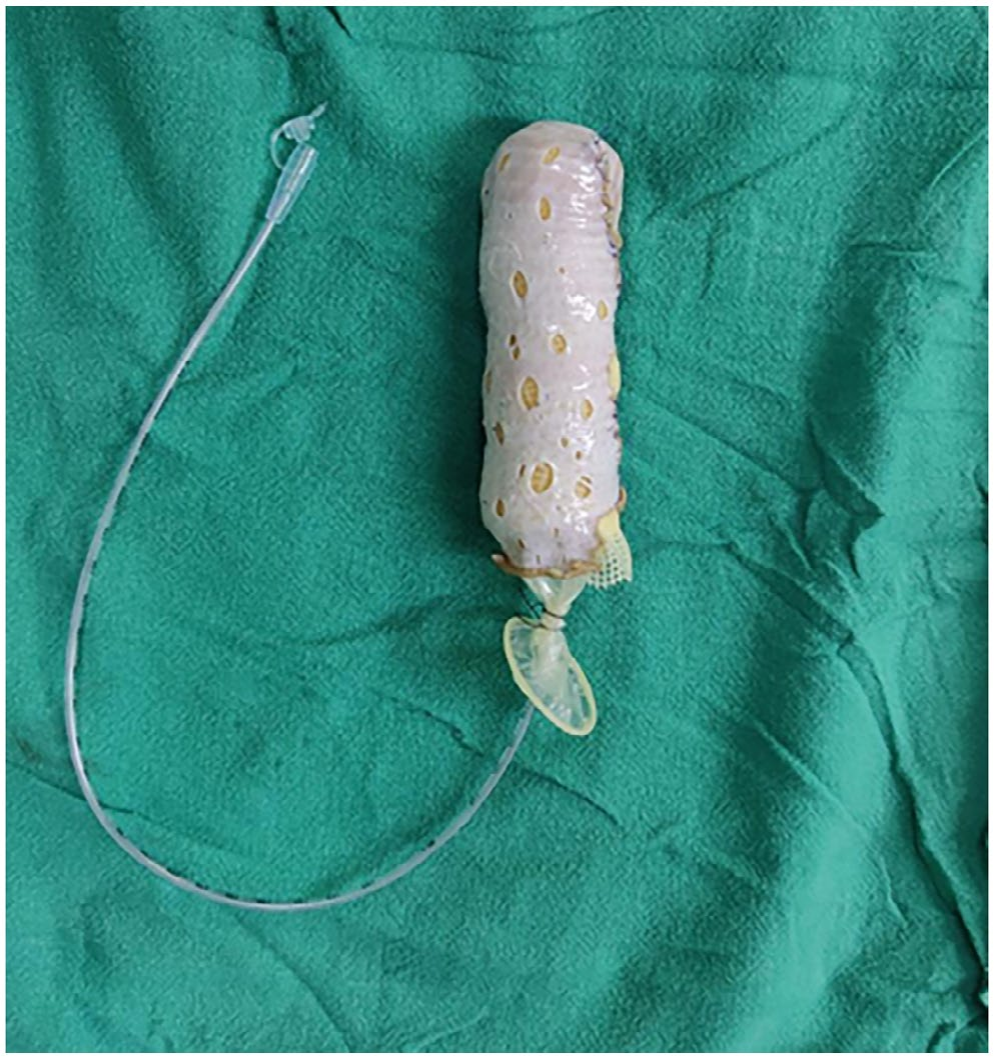
Vaginal mold wrapped with STSG.

A diagnosis of MRKHS type II was made. The patient was counseled about her condition. Surgery was planned for vaginoplasty to lengthen her vagina to a normal extent. A multidisciplinary team of gynecologists and plastic surgeons planned for McIndoe vaginoplasty. A subarachnoid block was administered, and a split‐thickness skin graft (STSG) was harvested from the anterior aspect of the right thigh. Preoperatively, a blind vagina approximately 2 cm in length was observed. A transverse incision was made over the dimpling area, followed by blunt dissection to create space on either side of the vestibule. A neovagina was then created, avoiding cystotomy and enterotomy. A vaginal mold was crafted using foam and an 8 Fr infant feeding tube, which was sealed with a condom (Figure 2). The mold was then wrapped with the STSG and inserted into the neovagina under negative pressure. Postoperatively, a 7‐cm neovagina was successfully created.

## 4 Conclusion and Results

Her skin graft site healed without issue. Subsequent hospital stays and follow‐ups were uneventful.

## 5 Discussion

The MRKHS was initially described by Mayer and subsequently by Rokitansky. Hauser and Schreiner later delineated the distinguishing features of MRKHS from androgen insensitivity syndrome (AIS) [2]. Mullerian duct abnormalities occur in 1% to 5% of women [5]. MRKHS is a rare congenital Mullerian duct abnormality characterized by the absence or underdevelopment of the uterus and parts of the vagina. However, the functions of the ovaries are typically preserved, along with normal secondary sexual characteristics. As cited by most articles, the overall incidence of this condition is approximately 1 in 4000 to 5000 women [2, 6, 7]. MRKHS is further categorized into two types: (i) type‐A MRKHS and (ii) type‐B MRKHS. Type‐A cases involve isolated uterine aplasia, whereas type‐B cases have both gynecological and non‐gynecological complexities like renal (30%–40%), skeletal (30%–40%), auditory deficit/deafness (10%–25%), and cardiac anomalies (4%). Our patient also had type B MRKHS, presenting with renal agenesis but without any cardiac or vertebral defects. Renal anomalies consists of unilateral renal agenesis (23%–28%), ectopia of one or both kidneys (17%), renal hypoplasia (4%), and a horseshoe kidney (5%) [1, 6, 7].

The exact etiology is unknown. However, it is believed that Rokitansky syndrome stems from a defective Müllerian fusion defect at the level of the urogenital sinus and vaginal plate during embryologic development. MRKHS is commonly identified in adolescent females who experience primary amenorrhea alongside the typical development of secondary sexual characteristics. It is the second most common cause of primary amenorrhea. Diagnosis of MRKHS mostly depends on imaging studies, trans abdominal USG is the first line of investigation in health facilities. MRI is the sole noninvasive modality with high sensitivity and specificity (100%) for diagnosing MRKHS [7]. Following ultrasonography, an abdominopelvic MRI examination should be conducted to assess for any associated defects in other organs (renal, vertebral, and cardiac) that may accompany this condition. Diagnostic laparoscopy can also be done to examine organs and complications such as endometriosis [1, 8]. Karyotyping seems normal phenotypically and genotypically, having an equal number of chromosomes (46XX). Besides normal karyotype, all other hormonal levels are also normal which correlates with our case [3]. The MRKHS should be differentiated from AIS and isolated vaginal hypoplasia or atresia which can be done by karyotyping studies. In AIS, a fetus with a male genotype (46XY) will experience resistance to androgen in their end organs. This resistance leads to the virilization of external genitalia, resulting in a baby with a female phenotype, including the development of female secondary sexual characteristics and undescended testes. Another distinguishing feature between MRKHS and AIS is the presence of normal serum levels of luteinizing hormone and follicular‐stimulating hormone, along with no signs of androgen excess [2].

The treatment objectives for MRKHS focuses on two key aspects: restoring normal sexual function and utilizing assisted reproduction techniques [5]. Creation of a neovagina can be pursued when the patient is interested in forming a vaginal canal for sexual activity and the patient is emotionally mature enough to be able to undergo the procedure and comply with postoperative maintenance. Neovagina creation can involve both surgical and nonsurgical approaches. In the nonsurgical method, known as the Frank's dilation method, vaginal dilators are placed on the perineal dimple for at least 20 min daily to increase the length and diameter of the vagina. This technique boasts a success rate ranging from 78% to 92% and is considered the primary therapy for neovaginal creation [9]. If nonsurgical methods prove ineffective, there are several surgical approaches available for neovagina formation. Some common techniques include sequential dilation using a traction device (known as the Vecchietti procedure), vaginoplasty with a skin graft (referred to as the McIndoe procedure), vaginoplasty with a peritoneal flap (known as the Davydov procedure), and colon vaginoplasty (typically performed using the sigmoid colon) [10]. The goal is to make a new vagina that is at least 8 in. long and 2 in. wide, with a lining of natural tissue, and does not require regular stretching for the rest of the person's life [9]. In a study of 86 McIndoe procedure patients (mean age: 21 ± 6 years; mean postsurgery duration: 23 ± 12 years), significant benefits were observed. 79% reported improved quality of life, 91% were sexually active (75% achieving orgasm), and 55% experienced enhanced self‐image. These findings underscore the procedure's positive impacts, particularly on sexual function and self‐perception [11].

The psychological effects of this syndrome include fears of being identified, concerns about sexuality, and coping with infertility. Diagnosis often occurs during adolescence, a period recognized for its sensitivity in both physical and emotional development. Patients with MRKHS encounter various challenges, beginning with clinical examinations of the affected organs, difficulties with penetrative sexual intercourse, and an inability to conceive [3]. The complications associated with MRKHS are not limited to vaginal agenesis but psychological distress as well. Hence, it underscores the importance of psychological counseling to the patient before any treatment.

## 6 Conclusion

MRKHS poses complex challenges in both diagnosis and management. Timely identification and comprehensive, multidisciplinary care are needed to effectively address this condition. Treatment modalities include surgical interventions such as neovaginoplasty, along with psychological counseling. Continuous research is needed to advance understanding and refine treatment approaches, enhancing outcomes and quality of life for the patients affected by MRKHS.

## Author Contributions

**Newton Ashish Shah:** conceptualization, data curation, software, writing – original draft, writing – review and editing. **Manish Yadav:** conceptualization, writing – original draft, writing – review and editing. **Suman Verma:** writing – review and editing. **Digraj Yadav:** conceptualization, writing – review and editing. **Aalok Kumar Yadav:** data curation, writing – review and editing. **Asmita Ghimire:** supervision, writing – review and editing. **Hima Rijal:** supervision, writing – review and editing. **Poonam Koirala:** conceptualization, supervision, writing – review and editing.

## Consent

Written informed consent was obtained from the patient for publication of this case report and any accompanying images. A copy of the written consent is available for review by the editor in chief of this journal on request.

## Conflicts of Interest

The authors declare no conflicts of interest.

## Data Availability

All the required information is within the manuscript itself.
